# Defying the Unfavorable Risk: The Efficacy of LDR Brachytherapy Monotherapy in Gleason 7 Prostate Cancer: A 16-Year Single-Center Retrospective Study

**DOI:** 10.3390/cancers18162546

**Published:** 2026-08-07

**Authors:** Carlos Rabaça, Domingos Roda, Guy Vieira, Bruno Pereira, Ricardo Godinho, Mário Lourenço, José Alberto Pereira, Margarida Regencio, Sofia Macedo, Rui Caetano Oliveira, Amilcar Sismeiro, Anabela Mota Pinto

**Affiliations:** 1 Center for the Treatment of Urological Diseases, R. Dom Manuel I n.° 8, Estádio Cidade de Coimbra, 3030-320 Coimbra, Portugal; domingos.roda@jcs.pt (D.R.); guy.p.vieira@gmail.com (G.V.); 4076@ipocoimbra.min-saude.pt (B.P.); 2243@ipocoimbra.min-saude.pt (R.G.); 3685@ipocoimbra.min-saude.pt (M.L.); 4023@ipocoimbra.min-saude.pt (J.A.P.); 3345@ipocoimbra.min-saude.pt (M.R.); sofia.capela.macedo@lusiadas.com (S.M.); geral@cetruc.pt (A.S.); 2 Portuguese Institute of Oncology, Av. Bissaya Barreto 98, 3000-075 Coimbra, Portugal; 3 Faculty of Medicine, University of Coimbra, Azinhaga de Santa Comba, Celas, 3000-548 Coimbra, Portugal; apinto@uc.pt; 4 Joaquim Chaves Saúde, Rua Aníbal Bettencourt, n.° 3, Edifício CORE, 2790-225 Carnaxide, Portugal; 5 Faculty of Health Sciences, University Beira Interior, Av. Infante D. Henrique, 6200-506 Covilhã, Portugal; 6 Hospital Lusíadas, Av. dos Hospitais Civis de Lisboa 8, 2724-002 Amadora, Portugal; 7 Institute of Histology and Embryology, Faculty of Medicine, University of Coimbra, Rua Larga, 3004-504 Coimbra, Portugal; rui.oliveira@uc.pt

**Keywords:** intermediate-disease-risk patients, ISUP 2, ISUP 3, low-dose-rate brachytherapy (LDR-BT), prostate cancer

## Abstract

**Background:** Prostate cancer patients with intermediate disease risk [Gleason score (GS)7] can be divided into two groups: GS = 7(3 + 4) (ISUP 2) (favorable disease risk) and GS = 7(4 + 3) (ISUP 3) (unfavorable disease risk). Low-dose-rate brachytherapy (LDR-BT) is an effective treatment for low- and intermediate-disease-risk patients. **Objectives:** The main goal of this study was to evaluate the effectiveness of LDR-BT monotherapy in prostate cancer patients classified as ISUP 3 when compared with ISUP 2 patients. **Methods:** This was a retrospective study that evaluated prostate cancer patients classified as ISUP 2 and ISUP 3 followed up at the Center for the Treatment of Urological Diseases, Portugal, who underwent LDR-BT. Overall survival (OS), biochemical recurrence-free survival (BRFS), bone metastasis and complications post-LDR-BT treatment were evaluated. **Results:** A total of 602 patients classified with GS = 7 (481/602 ISUP 2 and 121/602 ISUP 3) treated with LDR-BT were recruited. High OS rates and a reduced frequency of complications were described in both groups of patients. ISUP 3 patients had a lower BRFS rate, a higher risk of biochemical recurrence, and developed more bone metastasis in a shorter period when compared with ISUP 2 patients. No significant differences were detected in prostate-cancer-specific mortality between groups. **Conclusions:** LDR-BT monotherapy is a safe and effective treatment for prostate cancer patients with favorable and unfavorable intermediate disease risk, with similar OS rates and reduced complications.

## Simple Summary

Intermediate-risk prostate cancer includes patients with distinct prognoses, making it important to evaluate treatment outcomes according to disease characteristics. This study assessed the long-term effectiveness and safety of low-dose-rate brachytherapy used as the sole treatment in patients with favorable and unfavorable intermediate-risk prostate cancer. High overall survival rates and a low incidence of treatment-related complications were observed in both groups, supporting the safety and tolerability of this treatment approach. Patients with unfavorable intermediate-risk disease experienced biochemical recurrence more frequently and developed bone metastases earlier than those with favorable intermediate-risk disease. However, prostate-cancer-specific survival did not differ significantly between groups. These findings indicate that low-dose-rate brachytherapy monotherapy is an effective treatment option for both favorable and unfavorable intermediate-risk prostate cancer, while highlighting differences in disease progression that may require closer follow-up. This study provides clinically relevant evidence to support treatment selection, patient counseling, and future risk-adapted management strategies.

## 1. Introduction

Prostate cancer is the second most common malignancy diagnosed in men worldwide [1,2]. Approximately 1.5 million new cases and nearly 400,000 deaths are estimated globally each year [1,3].

The histopathological analysis of the prostate allows tumor differentiation grade according to the Gleason grading system, based in microscopic tumor cells appearance classified into five grades or patterns, numbered 1–5. The Gleason score (GS) is the sum of the most prominent and second most prominent Gleason pattern numbers, ranging from 2 to 10, and it can be used to categorize prostate cancers in grade groups: GS ≤ 6 (low grade cancer); GS = 7 (intermediate grade cancer); and GS ≥ 8 (high grade cancer). In 2014, a new grading system for prostate cancer was proposed by the International Society of Urological Pathology (ISUP) that grades cancer between 1 and 5 depending on the GS [4]. Gleason scores ≤ 6, 3 + 4 = 7, 4 + 3 = 7, 8 and 9–10 correspond to ISUP grades 1–5, respectively.

Brachytherapy (BT), using low-dose-rate (LDR) permanent seed implantation or high-dose-rate temporary source implantation, is an effective treatment option for selected patients with prostate cancer of any risk group [5,6,7].

The use of BT for the treatment of localized prostate cancer has markedly increased in Portugal over the past 25 years [8]. Despite its growing adoption, evidence regarding its clinical outcomes remains limited. Our group recently demonstrated that BT is an effective treatment for localized prostate cancer, achieving overall survival (OS) and biochemical recurrence-free survival (BRFS) outcomes comparable to those reported for radical prostatectomy and external beam radiotherapy (EBRT), while being associated with a lower rate of treatment-related complications [9].

Based on these findings, we hypothesized that low-dose-rate brachytherapy (LDR-BT) as monotherapy is an effective treatment option for patients with both ISUP 2 and ISUP 3 prostate cancer. Therefore, the aim of the present study was to evaluate the effectiveness of LDR-BT monotherapy in patients with localized ISUP 3 prostate cancer by comparing their oncological outcomes with those of patients classified as ISUP 2.

## 2. Materials and Methods

### 2.1. Patient Recruitment

This retrospective study included patients with localized prostate cancer classified as ISUP 2 or ISUP 3 who underwent low-dose-rate brachytherapy (LDR-BT) at the Center for the Treatment of Urological Diseases (Coimbra, Portugal) between 13 November 2007 and 18 March 2024. The study population represents a subgroup of a previously reported cohort from our institution [9].

Clinical and pathological data were retrospectively collected from medical records, including age, serum prostate-specific antigen (PSA) levels at diagnosis, clinical tumor (cT) stage, and D’Amico risk classification. Biochemical recurrence (BCR) was defined according to the Phoenix Consensus criteria as a PSA increase of ≥2 ng/mL above the post-treatment PSA nadir. During follow-up, data on overall survival (OS), biochemical recurrence-free survival (BRFS), development of bone metastases, and treatment-related complications following LDR-BT were also recorded.

Clinically relevant complications most commonly associated with LDR-BT were assessed as binary clinical endpoints (present or absent), rather than being graded according to a severity scale. Specifically, lower urinary tract symptoms (LUTSs) were considered a complication only in patients who did not present with these symptoms at diagnosis but developed them following LDR-BT, with persistence for more than one year after treatment and requiring medical therapy. Erectile dysfunction (ED) was considered treatment-related if it developed within the first five years after LDR-BT and required pharmacological treatment. In addition, acute urinary retention was defined as any episode requiring bladder catheterization, while radiation proctitis was recorded only when rectal bleeding occurred and required medical or procedural treatment.

All patients included in this study had localized prostate cancer at the time of treatment and were considered candidates for definitive LDR-BT. Before treatment, all patients underwent staging according to the international guidelines in force at the time of diagnosis. This included thoraco-abdominal-pelvic computed tomography (CT) and bone scintigraphy whenever indicated according to the patient’s risk profile. Treatment was initiated in patients with localized disease, without evidence of distant metastatic spread. None of the patients included in this study received neoadjuvant, concomitant, or adjuvant androgen deprivation therapy (ADT). All patients were treated with LDR-BT monotherapy, irrespective of ISUP classification.

### 2.2. Low-Dose-Rate (LDR) Brachytherapy (BT)

For all patients, irrespective of prostate volume, permanent seed implantation was performed using Iodine-125 (^125^I) with a prescribed dose of 145 Gy. The post-implant dosimetric evaluation was identical for both cohorts and followed the same institutional quality criteria: (a) prostate: D90 < 115% of the prescribed dose and V150 < 50%; (b) urethra: D30 < 150% of the prescribed dose; (c) rectum: V100 < 1.3 cc. These dosimetric objectives were applied uniformly to both cohorts, ensuring comparable treatment quality and allowing a valid comparison of oncological and functional outcomes.

### 2.3. Histopathological Evaluation

Formalin-fixed, paraffin-embedded (FFPE) prostate tissue specimens were routinely processed, and representative tissue sections were stained with hematoxylin and eosin (H&E) for histopathological evaluation. Histological assessment was performed by experienced genitourinary pathologists according to the recommendations of the International Society of Urological Pathology (ISUP). Prostate adenocarcinomas were assigned to ISUP Grade Groups based on the architectural patterns identified in H&E-stained sections, according to the current Gleason grading system criteria. Representative cases classified as ISUP Grade Group 2 and ISUP Grade Group 3 were selected for further analysis.

### 2.4. Immunohistochemistry

Immunohistochemistry was performed on representative FFPE tissue sections from selected ISUP Grade Group 2 prostate adenocarcinoma cases. Sections were stained with antibodies against α-methylacyl-CoA racemase (AMACR) and high-molecular-weight cytokeratin (34βE12) using standard immunohistochemical protocols. These markers were used as ancillary tools to support the diagnosis in morphologically equivocal cases. Immunohistochemistry was not routinely performed in ISUP Grade Group 3 tumors, as the diagnosis was established based on unequivocal histomorphological features on H&E-stained sections. Immunohistochemical findings were not considered for tumor grading, as ISUP Grade Groups were established exclusively based on the histomorphological evaluation of H&E-stained sections.

**Ethical considerations:** This study was performed in accordance with the ethical standards of the Declaration of Helsinki, and its subsequent amendments, and it was approved by the Ethics Committee of Faculty of Medicine, Coimbra, Portugal.

**Statistical analysis:** Survival curves were calculated using the Kaplan–Meier method and compared with the Log-Rank test. Cox proportional hazards regression analysis was performed for comparisons between groups and identification of prognostic factors. Comparisons between two independent groups were performed using the Mann–Whitney U test for continuous variables and the Pearson Chi-Square test for categorical variables presented in contingency tables. All statistical analyses were performed using IBM SPSS Statistics 31.0.1.0. Statistical significance was set at *p* < 0.05.

## 3. Results

### 3.1. Clinical Characterization of Patients

**Figure 1:**
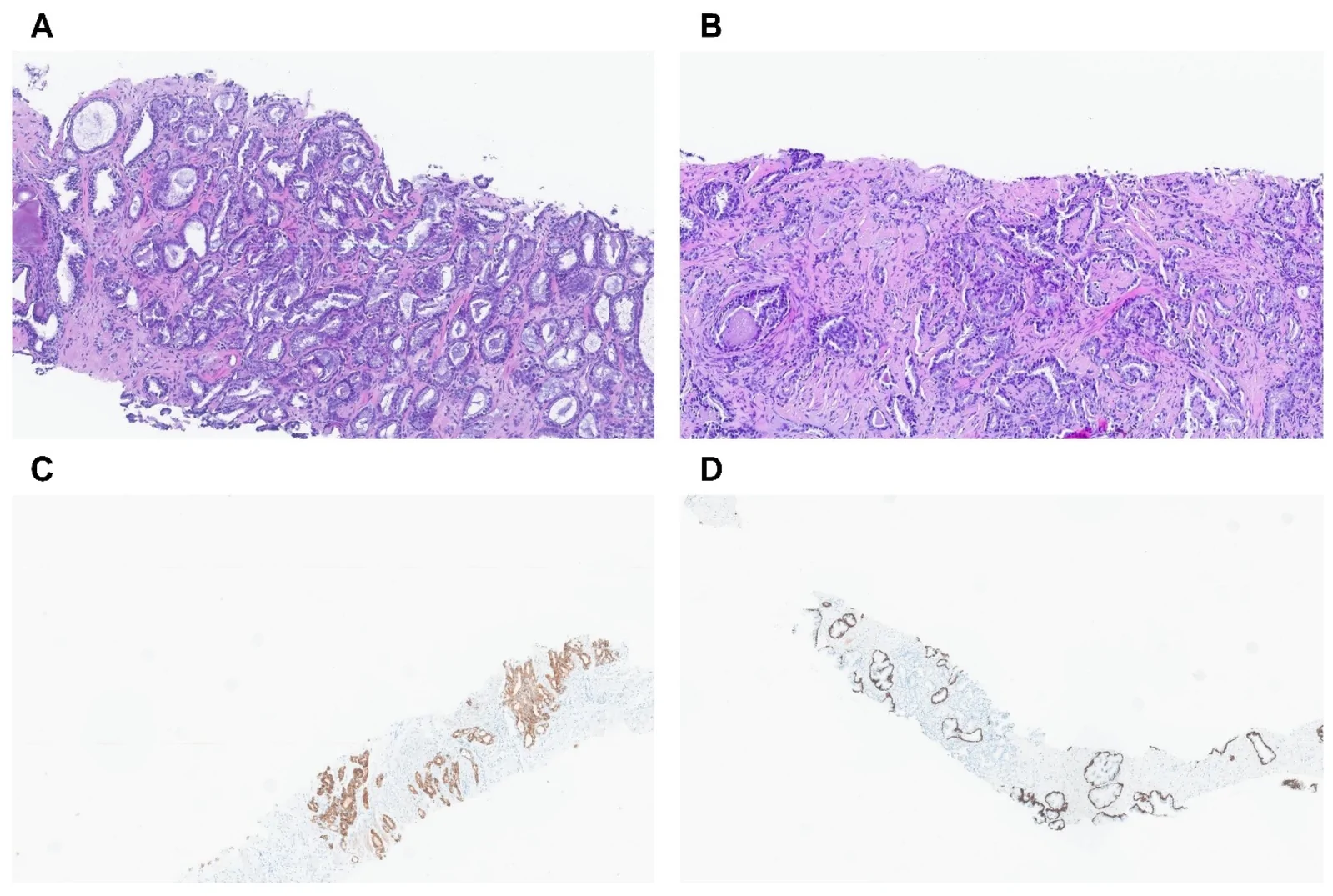
Representative histological and immunohistochemical images of prostate adenocarcinoma specimens classified as ISUP Grade Group 2 and ISUP Grade Group 3. (**A**) A hematoxylin and eosin (H&E)-stained section of an ISUP Grade Group 2 tumor. (**B**) An H&E-stained section of an ISUP Grade Group 3 tumor. (**C**) Representative immunohistochemical staining of an ISUP Grade Group 2 tumor showing α-methylacyl-CoA racemase (AMACR) overexpression. (**D**) Representative immunohistochemical staining of an ISUP Grade Group 2 tumor showing loss of 34βE12 expression.

A total of 602 prostate cancer patients classified with a GS = 7 who underwent LDR-BT were included. Patients were subdivided into two groups: (1) patients classified as ISUP 2 (N = 481, 80%); and (2) patients classified as ISUP 3 (N = 121, 20%). Representative histological and immunohistochemical images of prostate adenocarcinoma specimens classified as ISUP Grade Group 2 and ISUP Grade Group 3 are indicated in Figure 1.

Patients classified as ISUP 2 (Group 1) had a median age of 69 years (range, 46–87) and a median prostate volume of 40 cc (range, 13–115). According to D’Amico risk stratification, 12 (2.5%) patients were classified as low-risk, 426 (90.3%) as intermediate-risk, and 34 (7.2%) as high-risk. The median baseline serum PSA level was 6.71 ng/mL (range, 0.87–156.0), with 366 (76.6%) patients presenting PSA levels < 10 ng/mL, 88 (18.4%) between 10 and 20 ng/mL, and 24 (5.0%) ≥ 20 ng/mL. Regarding clinical stage, 420 (88.2%) patients were classified as cT1–T2a, 35 (7.4%) as cT2b, and 21 (4.4%) as cT ≥ 2c. The median time to PSA nadir was 36 months (range, 0–156), whereas the median time to biochemical recurrence (BCR) was 3 years (range, 0.3–13). Prostate-cancer-specific mortality (PCSM) occurred in 8 patients (1.7%), and the median follow-up was 8 years.

Patients classified as ISUP 3 (Group 2) had a median age of 71 years (range, 40–91) and a median prostate volume of 43 cc (range, 15–93). Based on D’Amico risk classification, 3 (2.5%) patients were categorized as low-risk, 106 (88.3%) as intermediate-risk, and 11 (9.2%) as high-risk. The median baseline serum PSA level was 7.46 ng/mL (range, 1.05–38.6); 86 (71.7%) patients had PSA levels < 10 ng/mL, 27 (22.5%) had PSA levels between 10 and 20 ng/mL, and 7 (5.8%) had PSA levels ≥ 20 ng/mL. Clinical staging showed that 96 (79.3%) patients had cT1–T2a disease, 15 (12.4%) had cT2b disease, and 10 (8.3%) had cT ≥ 2c disease. The median time to PSA nadir was 18 months (range, 0–168), and the median time to BCR was 2 years (range, 0.3–12). PCSM was observed in 5 patients (4.1%), and the median follow-up duration was 7 years.

The clinical characterizations of the patients included in this study are described in Table 1.

Patients classified as ISUP 2 had a lower median age (Mann–Whitney U test, *p* = 0.010) and a higher frequency of patients classified as cT1–cT2a (Pearson Chi-Square test, *p* = 0.036) when compared with ISUP 3 patients (Table 1). No statistically significant differences were detected in PCSM between groups (Pearson Chi-Square test, *p* = 0.187) (Table 1).

### 3.2. Patients with GS = 7 Have a Similar Overall Survival After LDR-BT Irrespective of ISUP Classification

**Figure 2:**
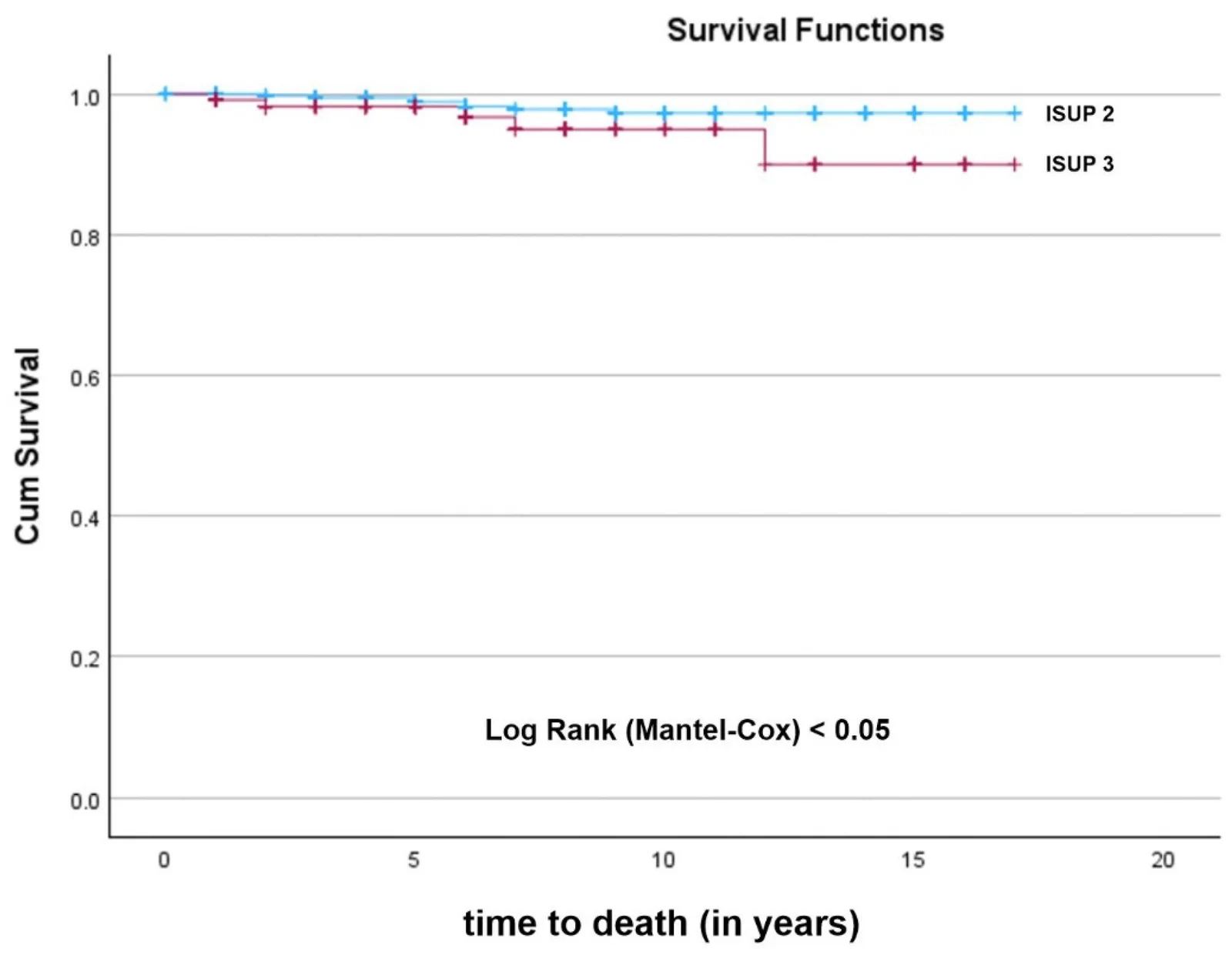
Kaplan–Meier curve of overall survival (OS) of prostate cancer patients classified as ISUP 2 and ISUP 3 after low-dose-rate brachytherapy (LDR-BT). Differences were considered statistically significant for *p* < 0.05.

OS rates were analyzed after LDR-BT in both groups of patients (Figure 2). No statistically significant differences were observed in OS rates between patients classified as ISUP 2 (98.3%) and ISUP 3 (95.9%) (Log-Rank test, *p* = 0.065) (Table 1).

### 3.3. ISUP 2 Patients Have a Higher Biochemical Recurrence-Free Survival (BRFS) Rate After LDR-BT When Compared with ISUP 3 Patients

Biochemical recurrence (BCR) was defined according to the Phoenix criteria as a post-treatment PSA increase of ≥2 ng/mL above the nadir value [10].

**Figure 3:**
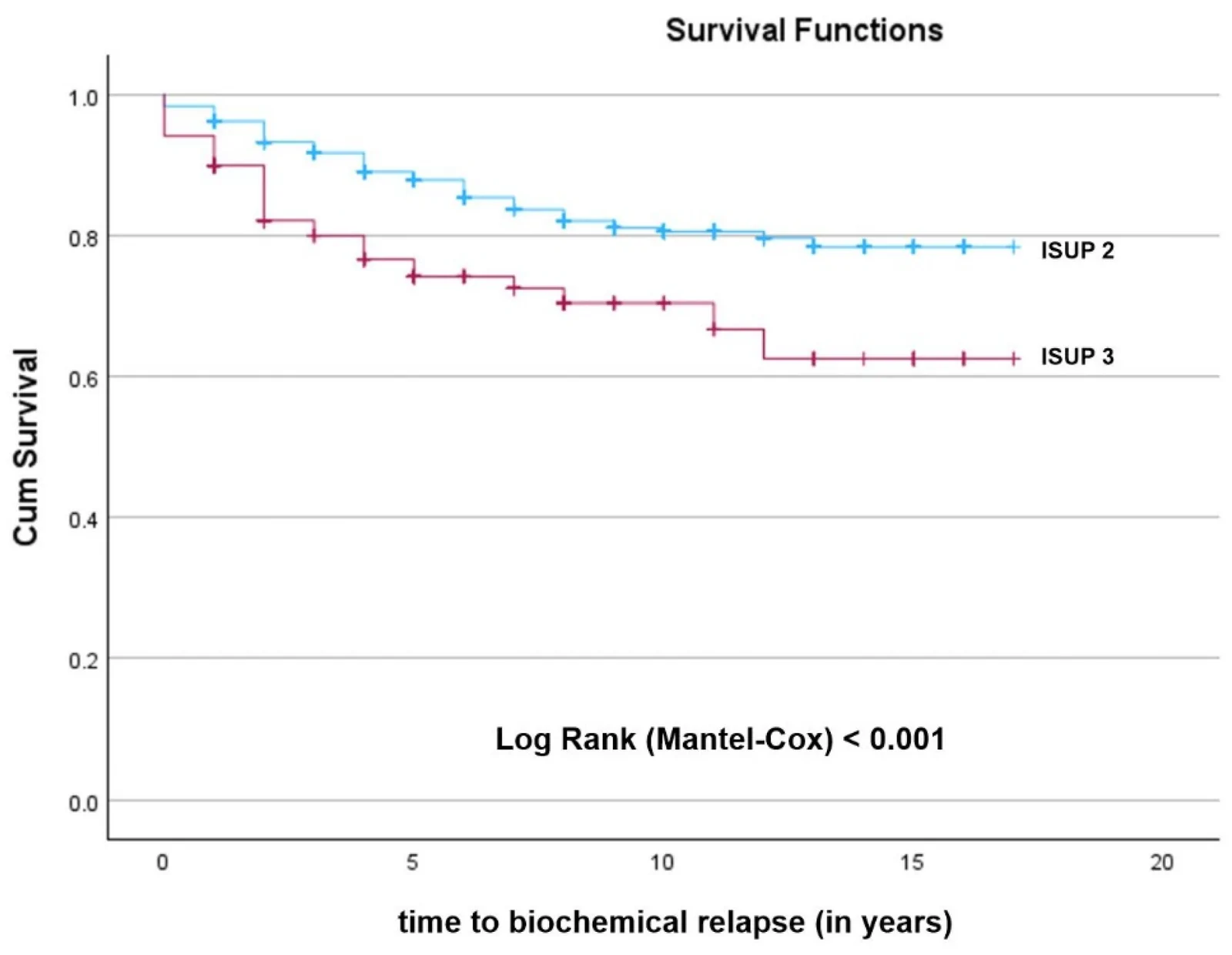
Kaplan–Meier curve of biochemical recurrence-free survival (BRFS) of prostate cancer patients classified as ISUP 2 and ISUP 3 after low-dose-rate brachytherapy (LDR-BT). Differences were considered statistically significant for *p* < 0.05.

BRFS rates were analyzed after LDR-BT in both groups of patients (Figure 3). Patients classified as ISUP 2 had a BRFS of 84.5% when compared with ISUP 3 patients (73.9%) (Log-Rank test, *p* < 0.001) (Figure 3 and Table 1). Multivariable Cox regression analysis showed that ISUP 3 patients had a significantly higher risk of biochemical recurrence than ISUP 2 patients (HR 2.007; 95% CI: 1.317–3.058, *p* = 0.001).

### 3.4. A Reduced Frequency of Complications Is Described After LDR-BT Irrespective of ISUP Classification

**Figure 4:**
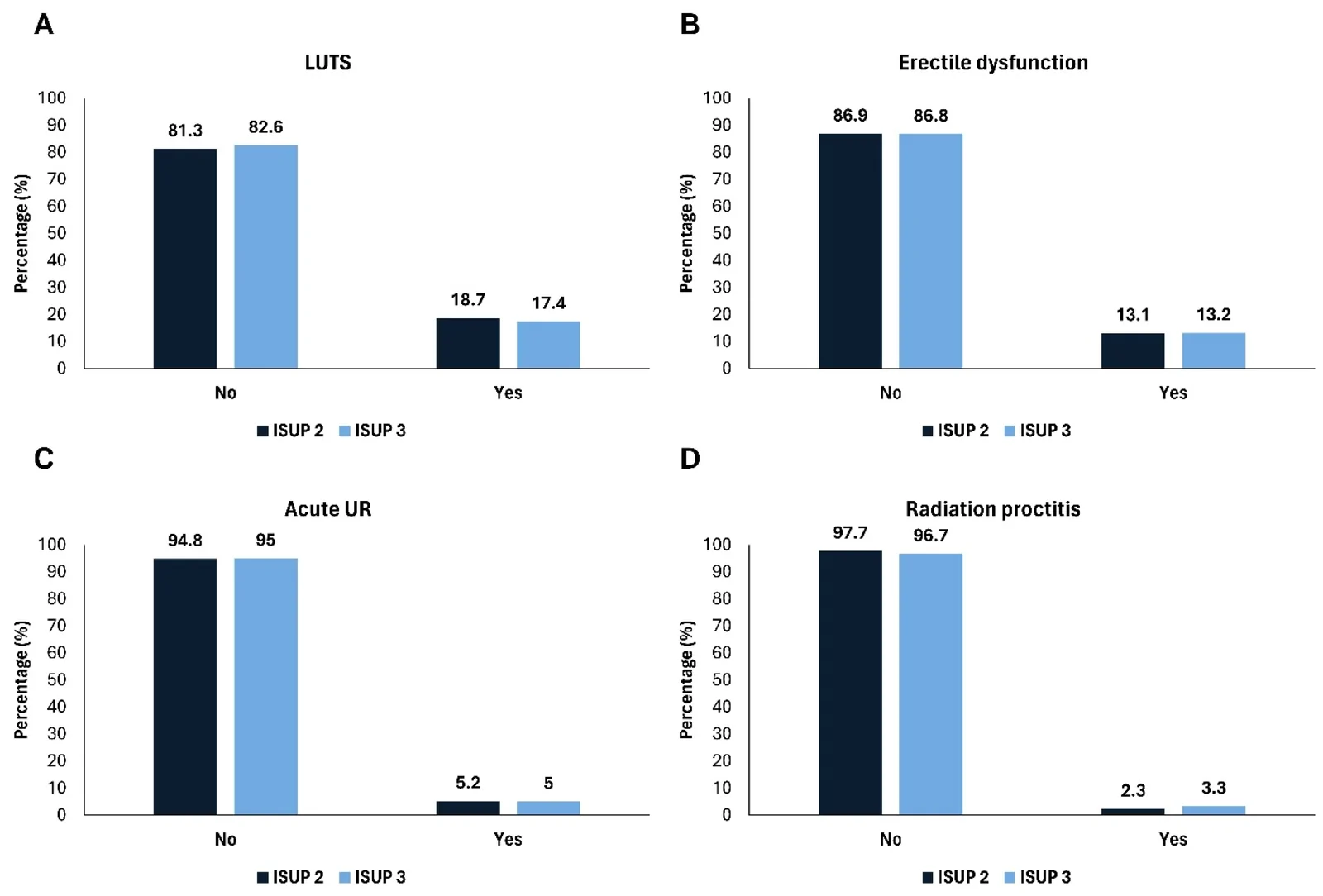
Complications described by prostate cancer patients classified as ISUP 2 and ISUP 3 after low-dose-rate brachytherapy (LDR-BT). Percentage of patients with lower urinary tract symptoms (LUTSs) (**A**); erectile dysfunction (ED) (**B**); acute urinary retention (UR) (**C**); and radiation proctitis (**D**) after LDR-BT. Pearson Chi-Square test was used for data analysis. Differences were considered statistically significant for *p* < 0.05.

Complications associated with LDR-BT treatment were described by a minority of patients with GS = 7 irrespective of ISUP classification, and no statistically significant differences were observed between groups (Figure 4). Lower urinary tract symptoms (LUTSs) were considered in patients who did not have this symptom during initial diagnosis, but who have developed it after LDR-BT and lasted more than one year post-treatment (Figure 4A). We found that 18.7% (N = 90) of ISUP 2 patients and 17.4% (N = 21) of ISUP 3 patients mentioned LUTSs after LDR-BT (Pearson Chi-Square test, *p* = 0.731) (Figure 4A). Erectile dysfunction (ED) was considered a complication if it occurred during the first 5 years after LDR-BT, and it was described by 13.1% (N = 63) of ISUP 2 patients and 13.2% (N = 16) of ISUP 3 patients (Pearson Chi-Square test, *p* = 0.971) (Figure 4B). Furthermore, 5.2% (N = 25) of ISUP 2 patients and 5.0% (N = 6) of ISUP 3 patients mentioned acute urinary retention (UR) after LDR-BT (Pearson Chi-Square test, *p* = 0.915) (Figure 4C). Radiation proctitis post-treatment was considered in symptomatic patients (rectorrhagia), and it was described by 2.3% (N = 11) of ISUP 2 patients and 3.3% (N = 4) of ISUP 3 patients (Pearson Chi-Square test, *p* = 0.520) (Figure 4D).

### 3.5. ISUP 3 Patients Developed More Bone Metastasis Post-Treatment in a Shorter Period of Time When Compared with ISUP 2 Patients

The frequency of bone metastasis after LDR-BT was also compared in both groups of patients. The median time until prostate cancer patients developed bone metastasis after LDR-BT was 6 years (range 0.3–11) and 3.5 years (range 0.6–13) for ISUP 2 and ISUP 3 patients, respectively (Mann–Whitney U test, *p* = 0.022, Table 1). Multivariable Cox regression analysis showed that ISUP 3 patients had a significantly higher risk of developing bone metastases after LDR-BT than ISUP 2 patients (HR 2.478; 95% CI: 1.292–4.750, *p* = 0.006).

**Figure 5:**
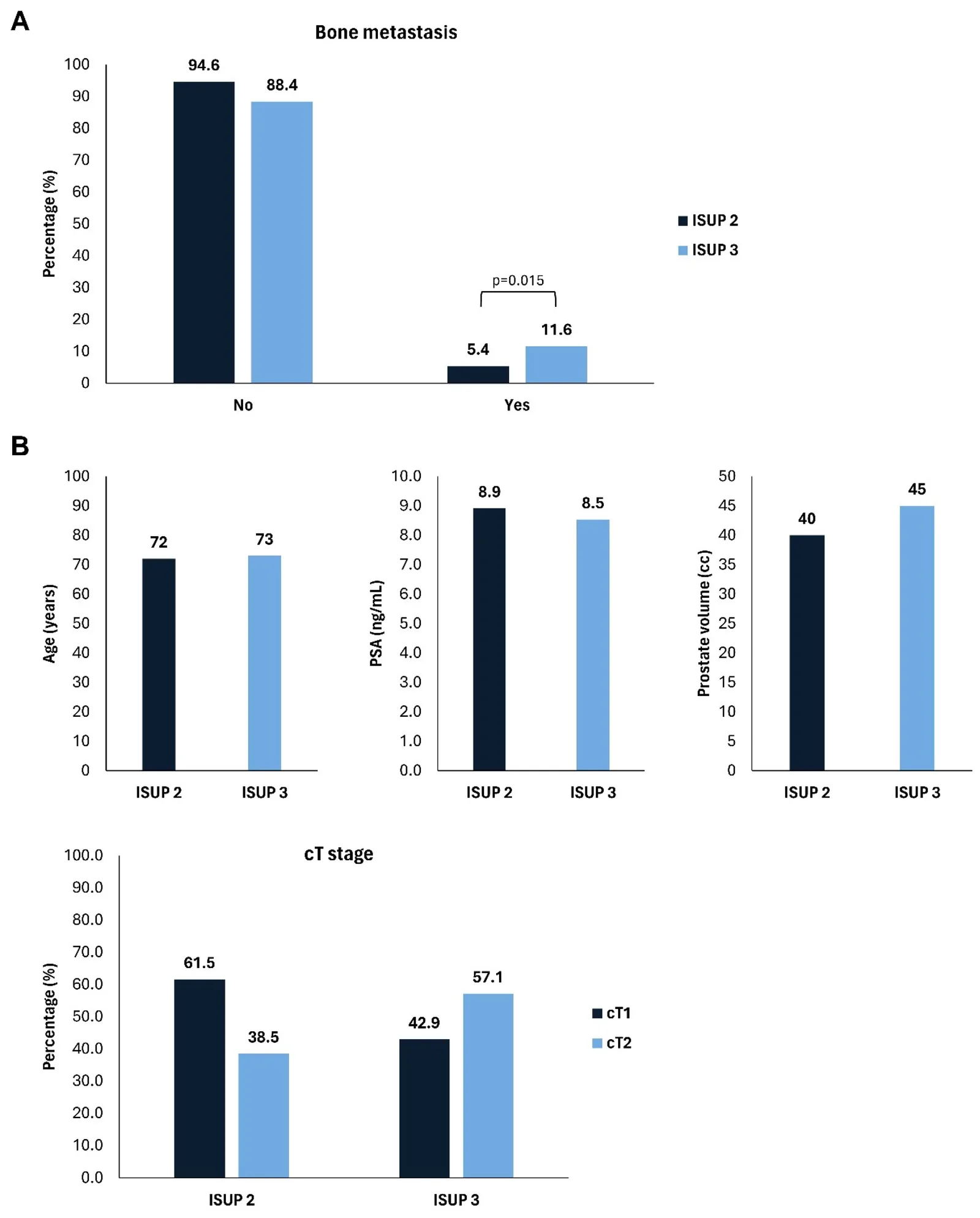
Bone metastasis after low-dose-rate brachytherapy (LDR-BT). Frequency of prostate cancer patients classified as ISUP 2 and ISUP 3 who have developed bone metastasis after LDR-BT (**A**); and comparison of baseline clinical data (median values of age, PSA, prostate volume and cT stage) between groups (**B**). Pearson Chi-Square test (bone metastasis and cT stage) and Mann–Whitney U test (age, PSA, prostate volume) were used for data analysis. Differences were considered statistically significant for *p* < 0.05.

From the initial cohort of patients classified with GS = 7 (N = 602), a total of 40 patients (6.6%) developed bone metastasis after LDR-BT, while most patients (N = 562, 93.4%) did not. It was observed that the frequency of ISUP 3 patients who developed bone metastasis after LDR-BT (14/121, 11.6%) was statistically significantly higher in comparison with ISUP 2 patients (26/481, 5.4%) (Pearson Chi-Square test, *p* = 0.015) (Figure 5A). No statistically significant differences were found between ISUP 2 and ISUP 3 patients who developed bone metastasis after LDR-BT regarding age (median values: 72 vs. 73 years, respectively; Mann–Whitney U test, *p* = 0.989), baseline PSA levels (median values: 8.91 vs. 8.56 ng/mL, respectively; Mann–Whitney U test, *p* = 0.176) and prostate volume values (median values: 40 vs. 45 cc, respectively; Mann–Whitney U test, *p* = 0.301) (Figure 5B). Among patients with bone metastases, the proportion of cT2 disease was higher in ISUP 3 patients (8/14, 57.1%) when compared with ISUP 2 (10/26, 38.5%); however, this difference was not statistically significant (Pearson Chi-Square test, *p* = 0.993) (Figure 5B).

## 4. Discussion

The distinction between ISUP 2 and ISUP 3 disease has become increasingly important in modern prostate cancer management, since predominant Gleason pattern 4 has consistently been associated with more aggressive tumor behavior, higher metastatic potential, increased risk of biochemical recurrence, and worse long-term oncological outcomes [4,11,12,13,14].

Several studies, including a recent study by our group, have shown that LDR-BT is an effective treatment for low- and intermediate-disease-risk patients with clinical outcomes comparable to those of radical prostatectomy and EBRT [9,13,15,16,17]. Of note, evidence from systematic reviews and propensity score-matched analyses suggests that LDR-BT monotherapy achieves biochemical control rates comparable to radical prostatectomy and EBRT in patients with intermediate-risk prostate cancer [18,19,20,21].

The present retrospective study evaluated the efficacy and safety of LDR-BT monotherapy in localized prostate cancer patients classified as GS = 7, comparing ISUP 2 and ISUP 3 patients followed up at a single center in Portugal. Our findings have demonstrated excellent OS outcomes after LDR-BT in both groups. Furthermore, treatment-related complications such as LUTS, ED, acute UR and radiation proctitis were relatively modest and did not significantly differ according to ISUP classification. Importantly, these findings support the favorable toxicity profile of LDR-BT irrespective of ISUP classification and are consistent with previous studies [6,9,22,23].

Although OS rates were similar between groups, ISUP 3 patients presented significantly lower BRFS rates, a higher incidence and an earlier onset of bone metastasis after treatment when compared with ISUP 2 patients. Indeed, although metastatic progression occurred in only a minority of patients overall, ISUP 3 patients were associated with a significantly higher frequency of bone metastasis when compared with ISUP 2 patients. These observations are consistent with previous reports in the literature which have shown that Gleason pattern 4 is associated with more aggressive disease and higher metastatic potential [11,12,24]. The shorter median time to PSA nadir and earlier BCR observed in ISUP 3 patients may also reflect the more aggressive biological behavior of tumors with predominant pattern 4 architecture. Histopathological and molecular studies have demonstrated that prostate cancers containing a predominant Gleason pattern 4 exhibit greater genomic instability, increased proliferative activity, and a higher likelihood of adverse pathological features such as extracapsular extension, seminal vesicle invasion, and metastatic progression [25,26,27,28].

Additional reports have shown that ISUP 3 patients have a worse cancer-specific survival than ISUP 2 patients [29]. In the present study, no significant differences were detected in PCSM after LDR-BT treatment between groups. These findings further support the safety profile of LDR-BT monotherapy irrespective of ISUP classification.

The role of LDR-BT within the treatment landscape for intermediate-risk prostate cancer was also importantly evaluated herein. Previous reports have shown that BT treatment provides oncological outcomes comparable or superior to EBRT or radical prostatectomy in selected patients [9,30,31,32]. ASCENDE-RT investigators led by Rodda et al. [33] reported superior biochemical progression-free survival with LDR-BT boost compared with EBRT boost in intermediate- and high-risk disease. These studies and ours reinforce the role of BT as an important definitive treatment modality in intermediate-disease-risk patients.

The current study also contributes with valuable real-world evidence from a Portuguese tertiary referral center over an extended follow-up period. Notably, it enabled the identification of clinically meaningful differences in metastatic progression between GS subgroups.

Some study limitations should be acknowledged. First, the retrospective single-center design inherently introduces potential selection bias and might limit causal inference. Second, the study spans a prolonged treatment period (2007–2024), during which diagnostic imaging, pathological grading, treatment planning, and supportive care evolved substantially. Additionally, the relatively smaller number of patients classified as ISUP 3 may have limited statistical power for some subgroup analyses, and, therefore, results should be carefully interpreted. Future studies ideally should incorporate additional parameters to further refine risk stratification among intermediate-risk prostate cancer patients treated with LDR-BT. Indeed, emerging evidence suggests that cribriform morphology, intraductal carcinoma, genomic instability signatures, and molecular biomarkers may improve prediction of recurrence and metastatic progression beyond conventional Gleason grading alone [34,35,36,37]. Prospective multicenter studies with standardized treatment protocols and centralized pathology review are warranted to validate these findings.

Overall, this study demonstrates that LDR-BT monotherapy is a safe and effective treatment for localized prostate cancer patients classified with GS = 7, irrespective of ISUP classification. Patients with ISUP 3 disease exhibit significantly worse biochemical control and increased metastatic progression in comparison with ISUP 2 patients, supporting the prognostic importance of predominant Gleason pattern and ISUP grading. These findings emphasize the biological heterogeneity within intermediate-risk prostate cancer and suggest that patients with ISUP 3 disease may benefit from closer follow-up, multimodal treatment strategies, and individualized risk-adapted therapeutic approaches. This is also consistent with clinical outcomes reported for EBRT and radical prostatectomy in similar patient populations [38,39,40]. Indeed, our findings suggest that, despite adequate local treatment, a subset of patients with predominant Gleason pattern 4 may harbor biologically more aggressive disease associated with occult systemic dissemination. Therefore, these results support further evaluation of treatment intensification in selected ISUP 3 patients with additional unfavorable intermediate-risk features. In this context, treatment intensification strategies, including the addition of short-course androgen deprivation therapy (ADT) and/or EBRT, may be considered in selected ISUP 3 patients. This interpretation is also consistent with contemporary risk-adapted treatment strategies, in which selected patients with unfavorable intermediate-risk disease are considered candidates for combined-modality therapy. Current clinical guidelines already recommend a multimodal approach for patients with unfavorable intermediate-risk prostate cancer, although the optimal selection criteria remain under investigation. Nonetheless, since the present study was retrospective and all patients were treated with LDR-BT monotherapy, it was not designed to determine whether combined treatment would improve oncological outcomes compared with monotherapy. Consequently, our findings should be regarded as hypothesis-generating and warrant prospective validation.

In summary, LDR-BT monotherapy represents a safe and effective treatment option for selected patients with localized GS = 7 prostate cancer. However, the inferior biochemical control and increased metastatic progression observed among ISUP 3 patients indicate that this subgroup represents a biologically distinct population that may benefit from individualized treatment selection. Rather than supporting a universal monotherapy approach, our findings emphasize the importance of careful risk stratification and suggest that selected ISUP 3 patients may benefit from treatment escalation, closer surveillance, and personalized multimodal therapeutic strategies. Prospective studies are needed to identify which patients derive the greatest benefit from combined treatment approaches.

To the best of our knowledge, this is the first study in Portugal that has analyzed the effects of LDR-BT monotherapy in a large cohort of prostate cancer patients with intermediate disease risk, comparing patients classified as ISUP 2 and ISUP 3, based on a single center analysis. Future studies with a multicenter national approach and collaborations are required.

## 5. Conclusions

LDR-BT monotherapy is a safe and effective treatment for localized prostate cancer patients with favorable and unfavorable intermediate disease risk, with similar OS rates and reduced complications post-treatment. Patients classified as ISUP 3 had a lower BRFS rate and developed more bone metastasis in a shorter period when compared to ISUP 2 patients, which supports the more aggressive behavior of Gleason score 4 pattern tumors and reinforces the usefulness of ISUP grading classification in disease prognosis. Nevertheless, our results suggest that LDR-BT monotherapy can be considered for the treatment of prostate cancer patients with GS = 7, irrespective of ISUP classification.

## Figures and Tables

**Table 1:** Clinical characterization of prostate cancer patients. Values represent median with range (minimum–maximum) and frequencies. Differences were considered statistically significant for *p* < 0.05. * Mann–Whitney U test; ** Log-Rank test; *** Pearson Chi-Square test.

Variable	ISUP 2 (N = 481)	ISUP 3 (N = 121)	*p* Value
Age (years)	69 (46–87)	71 (40–91)	*p* = 0.010 *
Follow-up time (years)	8 (1–17)	7 (1–17)	*p* = 0.101
Overall survival (%)	98.3	95.9	*p* = 0.065
BRFS (%)	84.5	73.9	*p* < 0.001 **
Prostate volume (cc)	40 (13–115)	43 (15–93)	*p* = 0.595
Time to PSA nadir (months)	36 (0–156)	18 (0–168)	*p* < 0.001 *
Time until BCR (years)	3.0 (0.3–13)	2.0 (0.3–12)	*p* < 0.001 *
Bone metastasis (%)	5.4	11.6	*p* = 0.015 ***
Time until bone metastasis (years)	6.0 (0.3–11)	3.5 (0.6–13)	*p* = 0.022 *
Prostate-cancer-specific mortality (%)	1.7	4.1	*p* = 0.187
**D’Amico classification (n, %)**			*p* = 0.762
Low-risk	12 (2.5%)	3 (2.5%)	
Intermediate-risk	426 (90.3%)	106 (88.3%)	
High-risk	34 (7.2%)	11 (9.2%)	
**PSA serum levels (ng/mL)**	6.71 (0.87–156.0)	7.46 (1.05–38.6)	0.571
PSA < 10 (n, %)	366 (76.6%)	86 (71.7%)	
PSA 10–20 (n, %)	88 (18.4%)	27 (22.5%)	
PSA > 20 (n, %)	24 (5.0%)	7 (5.8%)	
**Clinical tumor stage (cT) (n, %)**			0.036 ***
cT1–T2a	420 (88.2%)	96 (79.3%)	
cT = T2b	35 (7.4%)	15 (12.4%)	
cT ≥ 2c	21 (4.4%)	10 (8.3%)	

## Data Availability

The data presented in this study are available from the corresponding author on reasonable request.
